# Parents’ and Teachers’ Opinions about the School Food Policy in Belgian Flemish Nursery Schools

**DOI:** 10.3390/ijerph6031268

**Published:** 2009-03-24

**Authors:** Carine Vereecken, Hilde van Houte, Veerle Martens, Isabelle Wittebroodt, Lea Maes

**Affiliations:** 1 Department of Public Health, Ghent University, University Hospital BlokA, 2^nd^ floor, De Pintelaan 185, 9000 Gent, Belgium; E-Mail: Lea.Maes@UGent.Be; 2 Research Foundation Flanders (FWO); 3 Department of early childhood education, University College Arteveldehogeschool, Gent 9040, Belgium; E-Mails: hilde.vanhoute@arteveldehs.be (H-V.V.); veerle.martens@arteveldehs.be (V.M.); isabelle.wittebroodt@arteveldehs.be (I.W.); 4 Ghent University

**Keywords:** Preschool, school food policy, parents, teachers

## Abstract

The partnership of parents, teachers, and schools is necessary to develop effective school food interventions. To gather parents' and teachers' opinions and perceptions about the school food policy, 884 parents and 70 teachers of preschoolers completed a questionnaire. School food policy is an issue of importance for parents and teachers: the majority agrees that schools should restrict the availability of snacks and soft drinks; however, to replace fruit juice and sugared milk drinks with sugarless alternatives will take special effort. Fruit is not always available at school, although parents would appreciate it. Parents of lower educational level are in general more permissive.

## 1. Introduction

A recent study of Belgian Flemish preschool children shows that the diets served to many children do not meet the recommended daily intake of vegetables, fruit, cereals & bread, milk and fluids, while many children consume considerable amounts of sugared beverages and snacks [1], indicating that actions are needed to improve the children's diet.

In Flanders, more than 95% of the children 2.5–3 years old already go to school and consume their lunch and one or two snack meals at school five days a week. As such, schools are one of the best arenas to reach young children and their families for imparting nutrition education and should provide a context to promote healthy eating habits [2–4]. Moreover, evidence suggests that schools can make a difference [5,6] and school-based interventions can improve the dietary habits of children [7–10].

Policy responses are beginning to emerge and in January 2006, four Flemish Ministers signed a declaration of intent to initiate- and support health promotion measures in primary- and secondary schools (www.ond.vlaanderen.be/nieuws/2006p/files/intentieverklaring-26-01-2006.pdf, downloaded 15 Aug 2008). Additionally, the Minister of Education urged schools to have a school food policy by September 2007; 2007–2008 was considered a transitional year, a period for analysing the school environment and classroom activities and to take appropriate action.

However, to develop effective interventions, partnership among parents, the school staff, the community, and health professionals is needed. Effective programs must be tailored to community needs and take into consideration factors concerning individuals such as cultural background and equity aspects [11]. An important component in the early stages of programme development is, therefore, identifying parents' and teachers' attitudes and perceptions of the school food policy.

A study-specific questionnaire seeking opinions about the school food policy was developed; descriptive results of the opinions of parents and teachers will be presented and compared in the present paper. In addition, differences in parents' opinions by social status (SES) (operationalized by parental education) are investigated as differences in food consumption and rearing practices by social status have been identified in previous studies among children and adolescents in Flanders [12–15]. First, we will describe the school food policy in terms of availability and restrictions at school, anno 2008.

## 2. Methods

### 2.1. Design

Data of principals and teachers were collected as part of the baseline survey of the FIFI-study (Familial Influences on Food Intake), a longitudinal study on young children's food habits and their primary socialization (Study 1) [16].

Data of parents was collected as part of the baseline survey of an intervention study (Beastly Healthy at School) to assist Belgian nursery schools in the implementation of a healthy school food policy (Study 2) [17].

#### Study 1: the FIFI-study: data of teachers and principals

Eighty schools in East- and West-Flanders, randomly selected from the school list provided by the Ministry Department of Education, were approached for participation. Forty-six schools and ten sub-departments agreed to participate. The principals were asked to fill in a short school food policy questionnaire and the teachers of the participating classes (n = 90) were asked to fill in a teachers' questionnaire. Data collection was carried out during January–April 2008. A more detailed description of the FIFI can be found elsewhere [16].

#### Study 2: Beastly Healthy at school: data of parents

Four hundred and three schools in East-Flanders were e-mailed asking them whether they would be willing to participate in an intervention study to promote healthy eating, bearing in mind that there was a 50% chance that they would be randomized to control condition: 40 schools agreed to participate. Sixteen schools (eight control and eight intervention) were randomly selected. All parents of the pupils of the selected schools (n = 1,432) were asked to fill in a questionnaire including their socio-demographic characteristics, items related to the school food policy, and a food frequency questionnaire. It was explicitly asked that the parent who spent most time with the child outside school completed the questionnaire. The data was collected in September 2006. The impact of the intervention study and a more detailed description of the study have been described elsewhere [17].

### 2.2. Material

#### 2.2.1. School food policy questionnaire

Principals were asked to indicate for a list of foods and beverages if they were available DAILY at school, during morning breaks, lunch and/or afternoon breaks. For a selection of items, they had to indicate whether they were “allowed”, “never allowed” or “occasionally allowed (e.g. on birthdays)”.

Concerning fruit availability, principals were asked: (1) if there was “a fruit day” at school (“no”, “yes”), (2) if fruit was available at school, not taking into account fruit offered as part of a meal (“no or less than once a week”, “once a week”, “2–3 days a week”, “daily or almost daily”), and (3) how often fruit was available as dessert for those who ordered a hot meal at school (“no hot meal offered at school”, “not or less than once a week”, “once a week” “2–3 days a week”, and “daily or almost daily”).

#### 2.2.2. Teachers' and parents' school food policy opinions questionnaire

The school food policy opinions questionnaire was developed by the authors (including a communication expert, a pedagogue, a psychologist, and a pharmacist) and covered a broad range of school-food policy related issues such as education, communication, restriction rules, availability of food, and satisfaction. For a detailed description of the items see Table 2 (the original questionnaire is in Dutch). Each item had to be responded on a 5-point scale ranging from completely disagree to completely agree. For the purpose of analysis the variables were dichotomized (completely agree/agree versus no opinion/disagree/completely disagree).

A test retest study, with a 4–6 day test retest interval, was done in a small convenience sample (acquaintances and colleagues with young children; n = 24). Test-retest Kappa statistics of the dichotomized variables ranged between 0.36 and 1.00 with an average of 0.74 (eight items = almost perfect agreement (> 0.80); nine items = substantial agreement (0.61–0.80); three items moderate agreement (0.41–0.60); one item = fair agreement (0.21–0.40)).

### 2.3. Analysis

Multilevel logistic regression analyses were carried out to investigate differences between mothers, fathers, and teachers completing the questionnaires and to investigate a potential association of parents' opinion with their education. For the latter, the education of the respondent was categorized into high = bachelor or master or low = secondary school or less; analyses controlled for the gender of the responding parent. Finally, associations of school food policy satisfaction with each of the opinions about the own school food policy were investigated.

We anticipated that our individual responses (the opinions about the school food policy) would be clustered by school; therefore, our parents and teachers at level 1 were nested within schools at level 2. The independent variables are presented as dummy indicator variables contrasted against a base category. P-values < 0.05 are considered significant. MLwiN software version 2.02 was used to calibrate the models using second order Predictive/Penalized Quasi-likelihood (PQL) approximation procedures.

## 3. Results

### 3.1. Participants

#### Study 1

Fifty principals completed the school questionnaire (89% of the participating schools). Of the 90 teachers approached, 70 returned (78%) a completed questionnaire. All teachers were women.

#### Study 2

Of the 1,432 children approached for participation, 884 (61.7%) returned a completed questionnaire: 84.8% were completed by mothers (M), 11.2% by fathers (F), the remaining 4% were completed by others or the information was lacking. Forty-eight and a half percent were boys, 50.6% girls, for 0.9% the information was missing. Parental education was as follows: mothers: 51.2 % low education, 46.2% high education, 2.6% missing; fathers: 55.3% low education, 37.4% high education, 7.2% missing.

### 3.2. Availability and Restrictions at Nursery Schools

Table 1 shows the daily availability of food and beverages in nursery schools. Most schools provide water, soup, fruit juice, and natural- and sugared milk beverages to the children. Soft drinks are available in only two of the 50 schools, in 35 schools soft drinks are not allowed, and in eight schools only occasionally. Sweets and crisps are only allowed for special occasions, and this even only in ten and six schools, respectively, whereas cake and pastry are not allowed in nine schools but occasionally allowed in 36 schools.

Forty-five of the 50 schools responded to have a “fruit day”. In 20 schools, fruit is available outside the regular meal for at least one day a week; in one school, fruit is available for at least 2–3 days, whereas in the remaining 29 schools (58%), in general, no fruit is available at the school. Of the schools offering a warm meal, 56% offer fruit as dessert at least once a week (13 schools once, six schools 2–3 times, and one school almost daily); 44% offer no fruit as dessert or offer it less than once a week.

### 3.3. Parents’ Opinions

Almost all parents (M: 98%; F: 94%) agreed that healthy food habits need to be initiated early in life and expected (M: 94%; F: 91%) that the school pays particular attention in helping the children acquire these healthy dietary behaviours (Table 2). Only a small percentage of the parents (M: 16%; F: 13%), think that the influence of parents is so great that schools cannot change the children's food intake, thereby indicating that parents think that the school can make a difference.

The majority of parents (M: 93%; F: 91%) like to receive information about what the child learns about physical activity and nutrition; nonetheless, only slightly more than half of the parents (M: 57%; F: 54%) think that parents should be involved in the school's food policy.

Seventy-nine percent find it a plus point if a piece of fruit is available daily at school. Most parents (M: 94%; F: 91%) would like that the teachers take care that the child drinks enough fluids; mothers more than fathers (M: 79%; F: 68%) like to be informed about what the child eats at school.

The majority (M: 83%; F: 78%), agrees that the school is allowed to put restrictions on what children bring to school as a snack; 70% of the mothers, but only 54% of the fathers, agree that soft drinks should be forbidden at nursery schools. On the other hand, only one-third agrees that drinks should be limited to natural milk, water, and soup.

Eighty-one percent of the mothers and 70% of the fathers consider themselves sufficiently informed about the school food policy. Slightly less (M: 76%; F: 67%) are satisfied with the school food policy; however, most others marked the mid-point, indicating they have no opinion (M: 20%; F: 30%), while only a few were dissatisfied (M: 4%; F: 3%). Only 62% of the mothers and 54% of the fathers are satisfied with the items available at school, again most others (M: 20%; F: 37%) did not have an opinion about it, while only 9% of mothers and fathers were dissatisfied with the food items available at school.

### 3.4. Teachers' Opinions

In general, the results of the teachers are quite comparable although some remarkable differences were found. Fewer teachers report that it is necessary to involve parents in the school food policy, that availability of fruit at school is a plus point, and that sweets should be allowed as a treat. More teachers are, however, satisfied with the food available at school, and more teachers think that the school is allowed to restrict what children bring to school as a snack, that soft drinks should be forbidden, and that parents are sufficiently informed about what their children learn at school.

### 3.5. SES Differences

The significant SES differences are reported in Table 3: SES differences are mainly related to food restrictions at school and educational aspects. Those of low educational level are less restrictive and find the role of the school in teaching a balanced diet less important. A reverse association, however, was found for learning new food items: those of low SES find it more important that children learn of new food items at school in comparison with their counterparts of high SES.

### 3.6. Associations between Several Aspects of the School’s Food Policy and School Food Policy Satisfaction

Table 4 shows strong positive associations between parents' satisfaction with the school's food policy and being informed about the policy, being satisfied with the available food items at school, being informed about their child's food and, physical activity learning activities, and their perception that teaching balanced dietary habits is important at their child's school.

## 4. Discussion

The purpose of the present paper was to investigate what preschool children's parents and teachers think about the school food policy in Flemish nursery schools. In international literature, only two studies on parents' and teachers' opinions about school food policy have been found: a qualitative study on parents, teachers, and school board members of Thai preschool children [18] and a quantitative study on parents and teachers of middle school students in the St-Paul-Minneapolis (MN, USA) metropolitan area [19]. In Victoria (Australia) lay people's view of children's food policies was investigated in a random sample of the population [20]. The present study is the first on this topic in a European country.

In agreement with previous findings [18,19,21], parents consider school food policy in general as important; moreover, they like to be informed about what happens at school and what their children consume. In general, parents and teachers agree that there should be a school policy restricting the consumption of snacks and soft drinks at school and in most schools, these foods are not allowed or only occasionally allowed. Fathers are slightly more permissive, especially regarding the soft drink consumption, which might be explained by the lower health consciousness of men [22,23].

Only about one-third of the parents in our study did agree, however, to restrict the beverages to natural milk, water and soup, indicating that a considerable number would like to have/keep fruit juice and/or sugared milk beverages on the school's beverages lists. On the one hand, these are an important source of vitamins and minerals [24], particularly for children, on the other hand there is evidence of an association between the consumption of sugared drinks (including some evidence for fruit juice) and obesity [25].

Our school questionnaire shows that fruit is not systematically available in Flemish nursery schools (58% not or less than once a week, 40% once a week); nevertheless, our findings indicate that most parents (79%) would consider availability of fruit at school as and advantage. Teachers are a little less keen on the availability of fruit at school. They possibly think more about the practical consequences of adopting such a policy: e.g., one has to manage to keep the fruit fresh, fruit often has to be peeled, and young children can easily make a mess of it. Moreover, teachers might feel that schools are not responsible for children's fruit and vegetable intake [26]. Nonetheless, 59% still agree that availability of fruit at school is an important plus point. Evidence in primary schools suggest that availability of fruit at school by subscription can increase consumption [27–29], although it must be said that availability of free fruit at school is more effective than subscribed schemes [30]. In Flanders (Belgium), the Tutti Fruttie project (http://www.fruit-op-school.be/) aims at increasing the consumption of fruit by weekly offering of fruit at school. The schools organize a weekly subscription program usually at a cost of 5–10 euros per year. Background information, games, contests, recipes, and other suggestions that can help in increasing fruit and vegetables consumption are also provided. Many schools who had already participated in this project and process evaluation made it clear that one of the success factors is the intersectoral collaboration between the profit-making- (fruit suppliers) and non-profit sectors (health promotion centres, school health centres, and schools) at the national- as well as local level. It was also recognized that providing fruit at low cost is the success factor for continuity; however, additional efforts should be put in for children of low SES parents [31].

Also worth mentioning is the difference in satisfaction between the parents and teachers; fewer parents are satisfied with the available food items (as already illustrated by the higher percentage who would consider daily availability as a plus point) and more teachers think that parents are sufficiently informed about their child's food and physical activity learning activities. Some caution, however, is necessary when comparing the data of parents and teachers as the data were collected in the context of two different studies for which different schools were approached.

Few differences are found between fathers and mothers. Fathers reported to be less informed; however, they also considered the matter as less important than mothers did. Fathers were also less restrictive, and this was reflected in the significantly higher percentage of fathers who would allow soft drinks. The latter agrees well with the findings of the study of Worsley [20] in which Australian lay people's views about the school food policy were investigated and some evidence was found for men being more tolerant than women.

In a previous study in preschool children in Flanders [12], mothers of lower education level were more permissive, in that they restricted fewer items than their counterparts of higher education level. Congruent therewith, we found that parents of lower education would restrict less food items at school level than parents of higher education. This shows the importance of schools in creating a context where healthy food choices and behaviours are promoted so that at least during school hours only healthy food items are available, and access to sweet- and savoury snacks is restricted. Therefore, policies supported by the different school authorities are needed whereby issues concerning food availability and all food related activities in schools can be tackled. In Belgium-Flanders, a platform has been created in which all actors (the school authorities, pupils, teachers, parents, centres for pupil counseling, health organizations, scientists, and politicians) are represented, in which these issues are discussed and converted into strategic- and operational plans. Additionally, more outreach to/education of lower SES parents may be necessary so that these parents are made aware of the reasons why schools are establishing healthier policies, and are thus more likely to cooperate and feel comfortable with it. Our results indicate that the school food policy is a salient issue for parents and teachers; in addition, parents are more likely to be satisfied if their children's dietary habits are an important point of interest at the school and are well informed about the school's food policy and what their child learns at school. This is important, as effective programs need to be supported by the parents and teachers. In addition, our results indicate that for some aspects (e.g. availability of fruit) there is further room for improvement.

## 5. Limitations of the Study

The data are from self-reports and hence responses might be subject to social desirability considerations, although there was no reason for parents and teachers to distort their opinions as they were provided with an envelope to return their completed questionnaires; in addition, the teachers responses were anonymous.

The schools included in the intervention study were recruited by e-mail. While the Internet offers a cheap- and quick way to contact many schools simultaneously, a disadvantage is, however, the low response rate (10% school response in the intervention study). A more personalized communication and follow-up by telephone usually leads to a higher response rate; however, this was considered not feasible because of limited time, staff, and budget resources.

Caution is necessary in generalizing the results, especially those concerning the satisfaction with their own school's food policy, bearing in mind that response from schools was low, that parents' opinions are based only on those of 16 schools, that only a small number of fathers participated in the study, that there might be a bias in selection of the parents and teachers participating in the study, and that the data of parents and teachers were collected in the context of two different studies for which different schools were approached.

## 6. Conclusions

Parents agree that schools should create a context for their children where healthy food choices and behaviours are promoted: while nutrition should be part of the curriculum, snacks and soft drinks should be restricted; however, to replace fruit juice and sugared milk drinks with sugarless alternatives would take special effort. Teachers are in general even more supportive to restrict less healthy food items. Parents would appreciate availability of fruit at school and like to be informed about what happens at school, including the dietary behaviour of their child. Parents of lower educational background are more likely to be more permissive (= would restrict less food items at school).

## Figures and Tables

**Table 1.** Availability of food and beverages at nursery schools in East- and West-Flanders, anno 2008 (n = 50).

	**Available daily at school**	
	n	%
Water (free, paid or both)	49	98
free	46	92
paid	16	32
Natural milk	42	84
Sugared milk drinks	38	76
Chocolate milk	38	76
Other sugared milk drinks	29	58
Yoghurt	8	16
Fruit juice	41	82
Sugared soft drinks	2	4
Diet soft drinks	1	2
Coffee/tea	5	10
Soup	44	88
Bread/sandwiches	5	10
Hot meal	38	76
Sweets	0	0

**Table 2.** Opinions and beliefs about the school food policy as reported by the parents and teachers of nursery schools, in Belgium Flanders: % agreeing and results of logistic regressions comparing mothers, fathers, and teachers.

	Mothers (n = 750) %	Fathers (n = 99) %	Teachers (n = 70) %	Fathers OR (95 % CI)	Teachers OR (95 % CI)
**General**					
healthy food habits need to be initiated early in life	98	94	-	**0.28 (0.10–0.76)**	
preschoolers influence each others food habits	75	77	81	1.10 (0.67–1.79)	1.40 (0.73–2.67)
the influence of parents on children's food habits is so great that the school can not change the children's food intake	16	13	24	0.81 (0.43–1.51)	1.59 (0.84–3.00)
**Education**					
the school should pay particular attention to helping children acquire healthy dietary habits	94	91	97	0.62 (0.29–1.31)	2.09 (0.50–8.81)
knowledge about a balanced diet should be imparted at school to preschool children	85	84	80	0.87 (0.49–1.56)	0.67 (0.35–1.29)
it is important that children learn about new foods at school	79	81	83	1.13 (0.66–1.93)	1.29 (0.64–2.61)
**Communication/involvement**					
parents should receive information about what their children learn at school about physical activity and nutrition	93	91	93	0.81 (0.38–1.71)	0.99 (0.37–2.66)
it is important that parents are informed about the content of the school's meals	84	77	81	0.64 (0.38–1.07)	0.80 (0.41–1.53)
parents should be involved in the school's food policy	57	54	34	0.85 (0.55–1.31)	**0.37 (0.21–0.64)**
**Food consumption at school**					
it is an important plus point that a piece of fruit is available at school daily	79	80	59	1.05 (0.61–1.81)	**0.38 (0.21–0.67)**
the teacher should take care that the children drink enough fluids during school hours	94	91	91	0.68 (0.32–1.48)	0.75 (0.29–1.96)
the school should inform the parents about what the child eats at school	79	68	71	**0.57 (0.36–0.92)**	0.63 (0.34–1.17)
**Food restrictions at school**					
the school is allowed to restrict what children bring along to school as snacks	83	78	97	0.71 (0.41–1.21)	**7.24 (1.55–33.84)**
soft drinks should be forbidden in nursery schools	70	54	88	**0.48 (0.31–0.75)**	**3.60 (1.59–8.14)**
nursery schools should allow only natural milk (not sugared), water, and soup	34	28	30	0.76 (0.47–1.25)	0.84 (0.43–1.65)
sweets should be allowed at school only as a treat	57	62	33	1.40 (0.90–2.20)	**0.36 (0.19–0.65)**
**Opinions about /satisfaction with own school food policy**					
teaching balanced dietary habits is an important point of interest at my child's/our school	78	72	88	0.74 (0.45–1.20)	2.15 (0.97–4.76)
I'm informed about the school food policy (rules and agreements about food at school).	81	70	-	**0.52 (0.32–0.85)**	
I'm/parents are sufficiently informed about my/their child's food and physical activity learning activities	63	55	78	0.71 (0.46–1.10)	**2.05 (1.06–3.97)**
I'm satisfied with the school's food policy	76	67	80	0.66 (0.41–1.07)	1.26 (0.63–2.54)
I'm satisfied about the food items available at school	62	54	74	0.73 (0.48–1.13)	**1.83 (1.00–3.33)**

OR (95% CI) for fathers and teachers with mothers as reference category; bold = significant OR.

**Table 3.** Significant differences in parents' opinions by educational level of the responding parent.

	High (n=393)	Low (n=441)	OR	95% CI
**Education**				
the school should pay particular attention to helping children acquire healthy dietary habits	96	92	0.53	(0.29–0.97)
knowledge about a balanced diet should be imparted at school to preschool children	88	82	0.57	(0.38–0.86)
it is important that children learn about new foods at school	76	82	1.42	(1.00–2.00)
**Communication/involvement**				
it is important that parents are informed about the content of the school's meals	87	79	0.56	(0.38–0.82)
**Food restrictions at school**				
the school is allowed to restrict what children bring along to school as snacks	91	75	0.31	(0.20–0.47)
soft drinks should be forbidden in nursery schools	82	56	0.29	(0.21–0.40)
nursery schools should allow only natural milk (not sugared), water, and soup	41	27	0.64	(0.46–0.89)
sweets should be allowed at school only as a treat	47	66	2.18	(1.62–2.94)

OR (95% CI) with high education as reference category, controlling for gender of parent completing the questionnaire.

**Table 4.** Significant results of logistic regression analyses: satisfaction with school food policy as dependent variable and opinions/satisfaction about own school food policy as independent variable.

**I'm satisfied with the school food policy**				
	Not satisfied^a^ % (n = 216)	Satisfied % (n = 634)	OR^b^	(95% CI)
Teaching balanced dietary habits is an important point of interest at my child's/our school	52	85	5.60	(3.92–7.99)
I'm informed about the school food policy (rules and agreements about food at school).	42	92	16.96	(11.24–25.6)
I'm/parents are sufficiently informed about my/their child's food and physical activity learning activities	41	69	3.12	(2.25–4.34)
I'm satisfied about the food items available at school	26	72	7.21	(5.03–10.35)

^a^ Not satisfied = those who did not agree = completely disagree, disagree and no opinion;

^b^ Separate analyses for each variable: reference categories: those who did not agree.
